# Elastography and Metalloproteinases in Patients at High Risk of Preterm Labor

**DOI:** 10.3390/jcm10173886

**Published:** 2021-08-29

**Authors:** Izabela Dymanowska-Dyjak, Aleksandra Stupak, Adrianna Kondracka, Tomasz Gęca, Arkadiusz Krzyżanowski, Anna Kwaśniewska

**Affiliations:** Department of Obstetrics and Pathology of Pregnancy, Medical University of Lublin, 20-081 Lublin, Poland; izabela.dyjak@gmail.com (I.D.-D.); adriannakondracka@wp.pl (A.K.); tomasz.geca@umlub.pl (T.G.); a_r_krzyzanowski@tlen.pl (A.K.); haniakwasniewska@gmail.com (A.K.)

**Keywords:** preterm labor, high-risk patients, ultrasound, elastography, metalloproteinases, MMP-8, MMP-9

## Abstract

Preterm birth (PTB) is the leading cause of perinatal morbidity and mortality. Its etiopathology is multifactorial; therefore, many of the tests contain the assessment of the biochemical factors and ultrasound evaluation of the cervix in patients at risk of preterm delivery. The study aimed at evaluating the socioeconomic data, ultrasound examinations with elastography, plasma concentrations of MMP-8 and MMP-9 metalloproteinases, and vaginal secretions in the control group as well as patients with threatened preterm delivery (high-risk patients). The study included 88 patients hospitalized in the Department of Obstetrics and Pregnancy Pathology, SPSK 1, in Lublin. Patients were qualified to the study group (50) with a transvaginal ultrasonography of cervical length (CL) ≤ 25 mm. The control group (38) were patients with a physiological course of pregnancy with CL > 25 mm. In the study group, the median length of the cervix was 17.49 mm. Elastographic parameters: strain and ratio were 0.20 and 0.83. In the control group, the median length of the cervix was 34.73 mm, while the strain and ratio were 0.20 and 1.23. In the study group, the concentration of MMP-8 in the serum and secretions of the cervix was on average 74.17 and 155.46 ng/mL, but in the control group, it was significantly lower, on average 58.49 and 94.19 ng/mL. The concentration of MMP-9 in both groups was on the same level. Evaluation of the cervical length and measurement of MMP-8 concentration are the methods of predicting preterm delivery in high-risk patients. The use of static elastography did not meet the criteria of a PTB marker.

## 1. Introduction

Premature birth is a crucial issue of modern perinatology. According to the World Health Organization, it is estimated that approximately 15 million children are born prematurely. One million of them die due to complications related to prematurity, and a large proportion has physical and mental disabilities [1]. In Poland, the percentage of premature births is close to the European average and amounts to approximately 6.7% of all live births (i.e., approximately 27,000 children). 

Preterm births have increased in the last 20 years. It results, inter alia, from a delay in the reproductive age of women giving birth for the first time and, therefore, a greater number of maternal health problems, such as diabetes, hypertension, infertility treatment, cancer [1]. The same data indicate a higher survival rate of premature babies, which is inextricably linked with an improvement in neonatal care in most countries. Currently, about 90% of babies born at 28 weeks of pregnancy have a chance of survival. Research conducted in Great Britain shows that the survival rate of newborns in the group between 22 and 23 weeks of gestation is approximately 51%, in 24 weeks of pregnancy—47%, and in 25 weeks—67% and half of the newborns born after 25 weeks of pregnancy develop normally [2]. The definition of preterm labor includes the criterion of the duration of pregnancy, and it is delivery after the 22nd week of pregnancy and before the 37th week of pregnancy [1].

The greatest risk of complications concerns children born up to the 33rd week of pregnancy. Therefore, it is important to identify risk factors for preterm labor, and thus qualify pregnant women to the group at risk of premature delivery [3]. These patients can be divided into two groups:

- High-risk group (these are patients who have had a history of preterm labor in previous pregnancies—about 15% of all preterm births).
- Low-risk group (85% of preterm deliveries without burdened medical history).

Preterm labor risk factors can be divided into maternal, environmental, occupational, and obstetric factors.

The maternal factors include:

- Low socioeconomic status;
- Black race (compared to the white race) [4];
- Low level of education;
- Age below 18 and over 40 [5];
- Free marital status;
- Low maternal body weight before pregnancy (BMI below 19) [6];
- Thyroid diseases and other maternal diseases, such as urinary tract infections and periodontal diseases [7].

The environmental and professional factors include:

- Environment pollution;
- Smoking and drugs addiction;
- Stress (doubling the risk of preterm labor) [8];
- Hard physical work, shift work, night work, and long-term standing [9].

The obstetric factors include:

- A history of preterm labor;
- Multiple pregnancies;
- Bleeding from the genital tract [10];
- Intrauterine infection;
- Endometriosis [11];
- Hypertension and diabetes;
- Incorrect amount of amniotic fluid;
- Abdominal surgery in the second and third trimesters of pregnancy.

The etiology of preterm labor is multifactorial. About 50% of premature deliveries are associated with spontaneous uterine contractions; in 20–30% of cases, it is the premature termination of pregnancy due to medical indications, while 20% of them are caused by the preterm premature rupture of membranes (PPROM). Romero, in 2006, proposed the so-called “common way of delivery”, which involves the activation of multiple systems that lead to biochemical and anatomical changes aimed at the expulsion of the fetus and postpartum [12]. According to the current state of knowledge, infections are the main cause of premature births. It is assumed that they are responsible for about 40% of premature births. The pathogens identified in the amniotic fluid include both aerobic and anaerobic bacteria and viruses. The cytokines involved in the activation of the “common pathway” in preterm labor are IL-1 and TNF-alpha. It has been shown that the concentration of IL-1 and IL-8 in the cervicovaginal secretion is significantly higher in women who gave birth prematurely [13]. In addition, IL-1 stimulates the production of prostaglandins by the temporal and amniotic fluid, thereby inducing uterine contractile activity. In the case of TNF-alpha, there are reports that the concentration of this substance is much higher in patients whose preterm labor is caused by rupture of the membranes [14]. It is related to the stimulation of the production of metalloproteinases, mainly MMP-1, MMP-2, and MMP-9, which, etiopathogenetically, are associated with premature rupture of the fetal bladder and maturation of the cervix [15]. Metalloproteinases are enzymes that catalyze the degradation processes of collagen types I, II, III, IV, VII, X, and elastin. Their activity in the membranes of the fetus is regulated by many different substances, including trypsin, elastase, and thrombin. Anti-inflammatory cytokines, such as IL-10, also play an important role in the mechanism that induces preterm labor and are involved in the immune recognition and maintenance of pregnancy.

The causes of preterm labor also include cervical insufficiency, which we define as painless opening and shortening of the cervix. The risk of premature childbirth is estimated at 75% when the assessment of the length of the cervix is below 25 mm in pregnant women before the 20th week of pregnancy [16]. There are also biochemical indicators of preterm labor; these include fetal fibronectin.

Ultrasound is one of the best methods of assessing the length of the cervical canal. During ultrasound examination of the cervix, the following parameters should be taken into account:

- Measurement of the length of the cervical canal;
- Assessment of the shape of the internal os and the cervical canal;
- Measurement of the internal os;
- Measurement of the length of the invagination of the fetal bladder;
- Cervical index;
- Assessment of the posterior angle of the cervix.

The total length of the cervix is the distance between the inner and outer os. Its functional length is the distance between the lower pole of the amniotic sac, to the cervix, and the external os. In practice, functional length assessment is important for the prediction of preterm labor. We use the TYVU (Trust Your Vaginal Ultrasound) scheme to evaluate the shape of the internal os. Incorrect values of the index for patients from the higher risk group significantly increase the real risk of preterm labor. According to the Fetal Medicine Foundation (FMF), in order to correctly assess the length of the cervical canal, certain conditions must be met [17]. The elastography technique was first described in 1991 by Ophir and his colleagues [18]. It is a method of imaging organs and tissues, assessing their stiffness and hardness. It determines the susceptibility of the examined tissue to compression and mechanical decompression. This is possible thanks to digital ultrasound and appropriate image processing obtained with a volumetric probe. Currently, the most advanced variant of this method is dynamic elastography (in other words, Shear Wave Elastography). It is based on the objective assessment and measurement of the stiffness of the tissue to obtain its numerical value expressed in kPa or m/s using the supersonic effect in the Mach cone and Young’s modulus (on the extensibility and compressibility of the media) [19].

Elastography is a modern diagnostic method that is based on the fact that the hardness of the tissue/organ changes significantly as a result of the disease process. There are attempts to predict preterm labor by assessment of placental flexibility. The rectus abdominis muscle and the subcutaneous tissue were used as reference points for the stiffness coefficients. In the case of the second factor, its value measured in the second trimester of pregnancy can be effectively used as a marker of preterm labor [20]. Metalloproteinases (MMPs) are proteolytic enzymes that contain a zinc ion in the catalytic center. Both the MMP-8 and MMP-9 enzymes are of crucial importance during labor. MMP-9 has also been identified in the amniotic fluid in patients at risk of preterm labor and in delivery patients [21]. There are reports on the relationship between ultrasound examination and the assessment of the concentration of MMP-9 as a marker of preterm labor within 7 days from the examination. Most importantly, the concentration of MMP-9 in the maternal blood plasma remains stable during uncomplicated preterm pregnancy until the onset of full-term labor. In active preterm labor, the concentration of MMP-9 is increased in the amniotic fluid. MMP-9 is concentrated and activated in the amniotic fluid during pregnancy complicated by premature rupture of the membranes. Moreover, in the case of inflammation of the membranes and PROM, the expression of the gene encoding MMP-9 is increased. Assuming a cut-off value of 15 mm for the measurement of the cervical length and a proMMP-9 concentration of 67.157 ng/mL in plasma, we can achieve similarly negative predictive values for preterm labor, approx. 96% for the length of the cervix and 96% for the concentration of pro-MMP-9, while for positive predictors, 69% and 60% respectively. Interestingly, when both prognostic factors were combined, the sensitivity and specificity of the test improved. However, in order to be able to use the combination of both tests to predict premature labor, studies on a larger group of patients are needed [15]. Increased concentration of MMP-8 is not only associated with the maturation of the cervix during labor but also in preterm labor, PPROM, and intrauterine infection. Increased concentrations of MMP-8 in the blood serum are associated with an increase in the number of leukocytes in vaginal swabs [22]. Neutrophils and the concentration of MMP-8 in the amniotic fluid are believed to be an inflammatory response to ascending vaginal infections [7,12,23,24,25].

As shown, preterm labor is a complex problem, and its diagnosis is one of the most important obstetric issues.

## 2. The Aim of the Study

The aim of this study was a biochemical and ultrasound evaluation of the cervix in patients at high-risk of preterm labor.

The aim of the work was achieved by assessing:

- Socioeconomic data;
- Ultrasound examinations with elastography;
- The concentration of MMP-8 and MMP-9 metalloproteinases in plasma and in vaginal secretions in the group of patients at risk of preterm labor compared to the control group of pregnant women with a physiological course of pregnancy.

## 3. Material and Methods

The clinical material of the study included 88 pregnant women hospitalized at the Department of Obstetrics and Pathology of Pregnancy of the Medical University of Lublin. Patients who met the criteria for high-risk premature labor between 25 and 38 weeks gestation qualified for the study group. The control group consisted of patients who did not report premature delivery symptoms without concomitant diseases. After learning about the purpose and method of conducting the research, all patients gave their informed and written consent to participate in this project. The consent for the research was issued by the Bioethics Committee at the Medical University of Lublin (KE-0254/134/2009 and KE-0254/294/2017).

**Figure 1:**
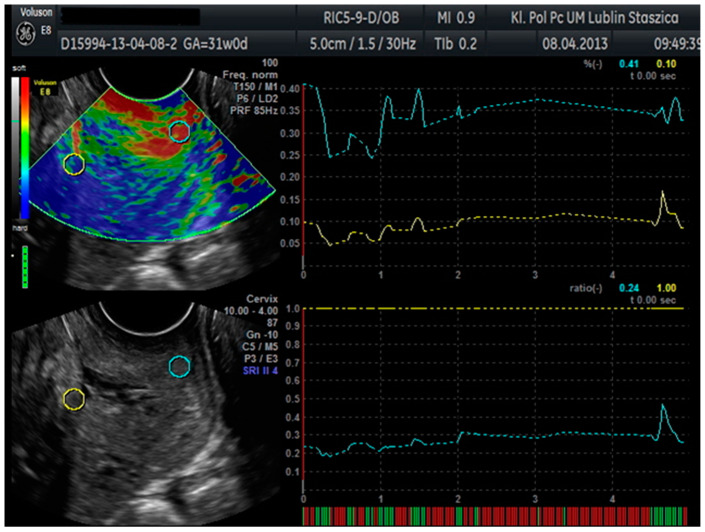
Cervical elastographic evaluation in the Elastography Analysis program.

In patients hospitalized at the Department of Obstetrics and Pathology of Pregnancy at the Medical University of Lublin with symptoms of premature labor, the diagnostic standard is an ultrasound examination, in which, in addition to the assessment of the fetal biometry and anatomy, the length of the cervix was determined. Blood samples were also collected from the patients included in the study, and a swab was taken from the posterior vaginal fornix during the gynecological examination preceding the ultrasound examination. The VolusonTM E8 with Elastography Analysis mode was used for the ultrasound examination. An endovaginal ultrasound was performed using an endoscopic probe (VolusonTM E8, RIC5-9-D). During the examination, the patients were asked to assume the lithotomy position. An endovaginal probe was placed in the anterior vaginal fornix, and the bladder was identified as an orientation point. A standard sagittal image of the cervix was then obtained, and the length of the cervix was measured. The probe, with elastography mode on, was used to produce up to five compression and decompression cycles. After confirming the correct compression and manual decompression in the form of a green quality bar in the lower-left corner of the screen, a measurement was made with each cycle lasting about 1 s. During each cycle, a tissue shift of approximately 1 cm was achieved. On the images obtained in this way, two regions were selected: area A on the upper cervical lip and area B on the bones of the fetal skull as the hardest reference point. Within these areas, circles of 5 mm in diameter were placed. From these circles, the Elastography Analysis program computed numerical values for the strain ratio and SR (SR–comparative tissue measurement). The value of the deformation factor means compression. The maximum value of compression in human tissue is 2%. The SR value, or comparative tissue measurement, indicates how much the tissue in the test area is harder or softer than the tissue in the reference test area. From these values, the means were calculated and used for statistical calculations (Figure 1).

**Figure 2:**
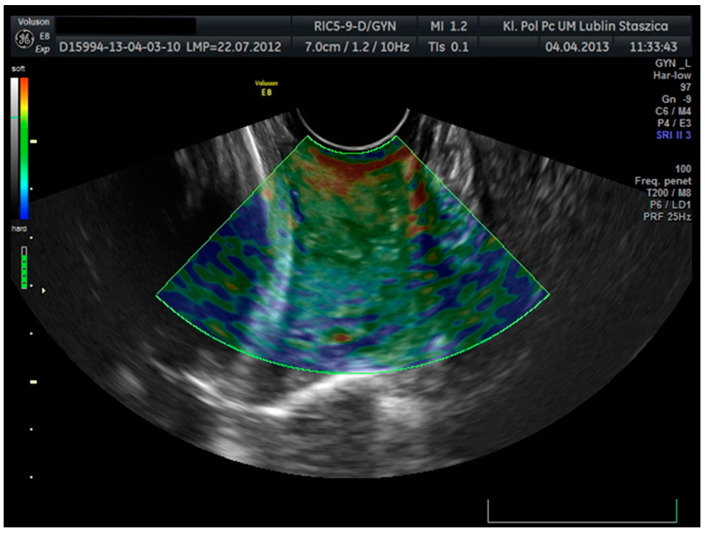
Cervical elastographic assessment. Tissue elasticity distribution calculations in real-time and presented using a color scale—red (soft), blue (hard), and green (medium-hard).

The transducer receives two sets of radiofrequency signals: before and after squeezing the cervix, and the amount of shift in the tissue is estimated from the waveform difference. Tissue rate versus distance from the transducer is calculated for all image points. The rate of change values are known as strain values and are displayed in a variety of colors ranging from red to yellow, green to blue for soft and hard tissues. Tissues marked in red are considered to be the most flexible tissues, while tissues with the least elasticity are marked in blue (Figure 2).

Laboratory tests were performed concurrently with the ultrasound examinations. These studies used serum samples separated from peripheral blood and samples of cervical secretions obtained from the simultaneous collection from a given patient.

Blood from the antecubital vein was collected in a volume of 9 mL into disposable S-Monovettes (Sarstedt, Germany) containing a blood clotting activator. The solidification process took place at room temperature and lasted approximately 30–40 min. The samples were then centrifuged in a centrifuge (Sigma 1-6P, Polygen) for 10 min at room temperature and 3800 rpm. The serum obtained in this way was aliquoted 200 µL in Eppendorf tubes (Medlab Products) and stored at −75 °C (Platinum Angelantoni 500, Italy) until the measurements were made.

Cervical discharge was collected with a sterile swab (Deltalab, Barcelona, Spain) while examined in a sterile speculum from the posterior vaginal fornix. If vaginal discharge could not be collected, a sample was collected from the cervix. A swab was inserted into the outer mouth of the cervix to a depth of 1–2 cm and then pressing the mucous membrane several times (for 10–15 s), which allowed for the absorption of the appropriate amount of secretion. The material collected in this way was placed in a sterile, tightly closed tube and sent to a laboratory.

In the next step, the swab with the sample was transferred to a tube containing 2 mL of PBS (Phosphate Buffered Saline (PAA Laboratories GmbH, Leonding, Austria) without calcium (Ca^2+^) and magnesium (Mg^2+^) ions). The secretion was extracted by vigorously rotating the swab inside the tube for about 10 s, and then the samples were centrifuged for 10 min at room temperature, at a speed of 3800 rpm. The supernatant thus obtained was collected from the sediment and aliquoted 200 µL. The material was stored in the same way as in the case of the serum.

Determination of the concentration of metalloproteinases 8 (MMP-8) and 9 (MMP-9) in the tested material was performed with the use of commercially available ELISA kits (Enzyme-linked Immunosorbent Assay) based on immunological reactions. All test steps were performed in accordance with the procedures recommended by the manufacturer of the assay kits. The following test was used to determine the concentration of MMP-8: Quantikine Human Total MMP-8 Immunoassay. For the quantitative determination of human active and pro-Matrix Metalloproteinase 8 (total MMP-8) concentrations in cell culture supernatants, serum, plasma, and saliva, Cat # DMP800 was used (R&D Systems Europe Ltd., Abingdon, UK). The mean analytical sensitivity of the assay was 0.02 ng/mL (0.01 to 0.06 ng/mL), and the measuring range was from 0 to 10 ng/mL (lowest concentration to highest standard concentration).

The following assay was used to determine the level of MMP-9: Quantikine Human MMP-9 Immunoassay. For the quantitative determination of human active (82 kDa) and Pro- (92 kDa) Matrix Metalloproteinase 9 (MMP-9) concentrations in cell culture supernatants, serum, plasma, saliva, and urine, Cat # DMP900 was used (R&D Systems Europe Ltd., Abingdon, UK).

The sensitivity of the assay, defined as the minimum detectable dose (MDD) of human MMP-9, was less than 0.156 ng/mL. The measuring range was from 0 to 20 ng/mL (from the lowest concentration to the highest standard concentration).

A statistical analysis of clinical data was performed using: the arithmetic mean standard deviation (SD), medians (ME) using the Shapiro–Wilk test, and the Kruskal-Wallis H test. A comparison of the differences between the control group and the study group was carried out with the Student’s *t*-test. For the variables tested, which did not show normal distribution, non-parametric tests were used for further analysis. The concentrations of MMP-8 and MMP-9 were compared using the U-Mann-Whitney test and the Pearson Chi2 test. The relationship between individual substances and clinical data was carried out using the Spearman correlation test. Statistical significance was set at *p* ≤ 0.05. Results statistically insignificant were defined by the abbreviation “ns”. Statistical calculations were based on Statistica 10 (StatSoft, Tulsa, OK, USA).

## 4. Results

### Characteristics of the Study and Control Groups

The study group included 58 pregnant women at risk of preterm labor, while the control group included 30 healthy pregnant women with a physiological course of pregnancy. Based on medical records, questionnaires, and an interview, demographic, social, and clinical data were collected for each patient participating in the study. The demographic, socio-clinical characteristics of the study and control groups are presented in Table 1, Table 2 and Table 3.

**Figure 3:**
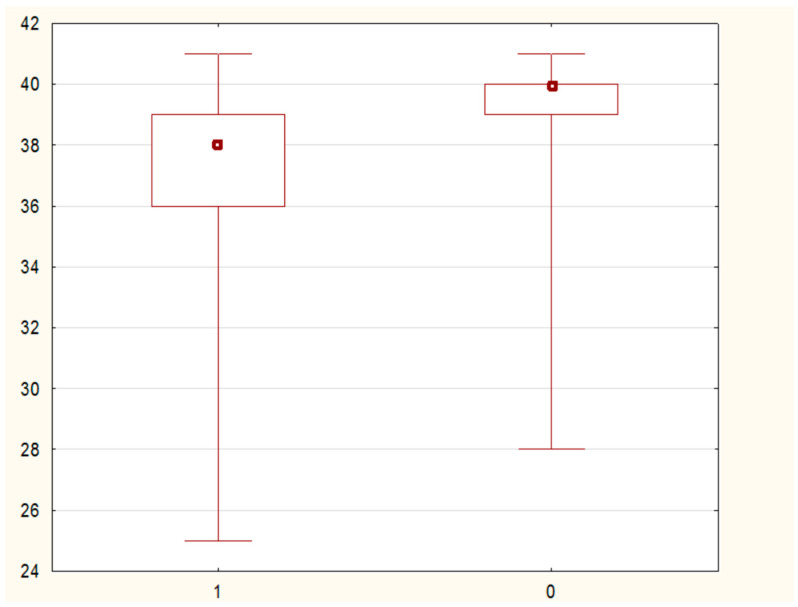
A box-whisker chart showing the comparison of the duration of pregnancy (wks) parameter in the study-1 and control-0 groups.

**Figure 4:**
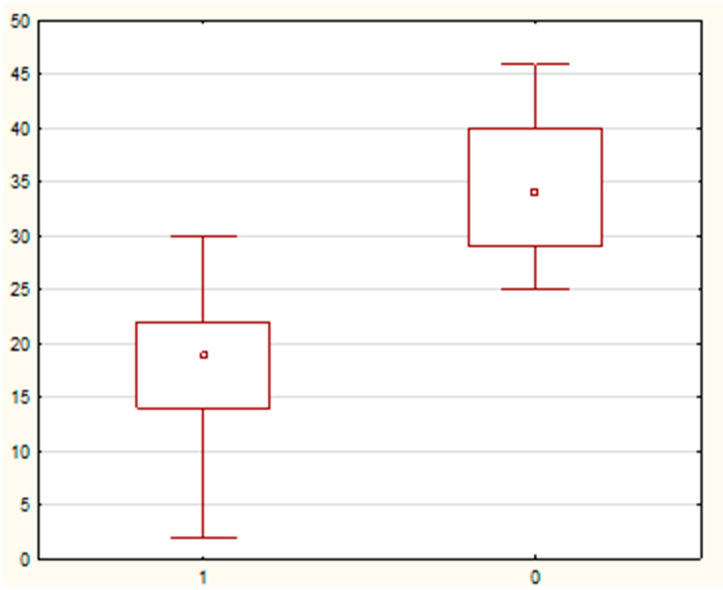
A box-whisker chart showing a comparison of the value of the variable cervical length—mm in the study-1 and control-0 groups.

The comparison of the distribution of values of selected demographic and clinical parameters in the study and control groups showed statistically significant differences in the variables—duration of pregnancy, CL, and MMP-8 concentration (assessed in samples of cervical secretion collected from the posterior vaginal fornix). It was noted that the parameter duration of pregnancy in weeks had significantly higher values in the control group than in the study group (40 (95% CI: 39–40) vs. 38 (95% CI: 37–39) weeks; test value U = 451 0; test value Z = −3.6; significance level *p* = 0.0002). Figure 3 shows a comparison of the parameter value the duration of pregnancy in weeks in the study and control groups. Similarly, the variable CL in mm had significantly higher values in the control group compared to the study group (34 (95% CI: 30, 17–39) vs. 19 (95% CI: 16–21) mm; test value U = 12.0; test value Z = −7.52; significance level *p* < 0.0001). In the case of the other examined variables, no statistically significant differences were found in the distribution of data. A graph comparing the value of the variable neck length in the test and control groups is presented in Figure 4.

**Figure 5:**
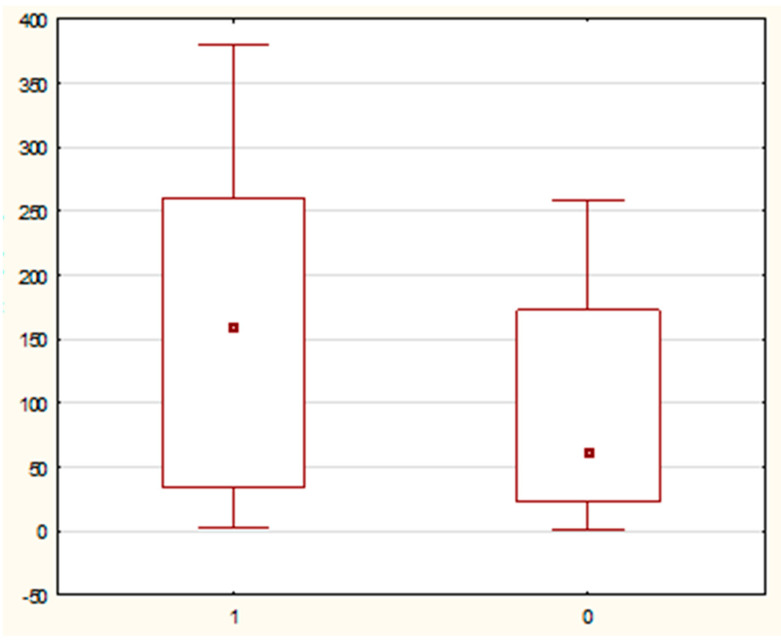
A box-whisker plot showing a comparison of MMP-8 ng/mL concentrations (assessed in cervical samples) in the study-1 and control-0 groups.

However, in the case of MMP-8 concentration significantly lower values of this parameter were observed in the control group compared to the study group (60.95 (95% CI: 25.38–89.86) vs. 158.34 (95% CI: 61.81–216.99) ng/mL; *U* test value = 631.5 Z test value = 1.99 and significance level *p* = 0.0459. Presented in Figure 5.

Based on the data analysis, several correlations were observed between the selected demographic and clinical parameters in the study group. There was a statistically significant mean negative correlation between the strain values and the SR-comparative tissue measurement (rho = −0.4248; *p* = 0.0010). Moreover, a statistically significant, average positive correlation was observed between the values of the cervix length and the parameter of pregnancy duration (wks) (rho = 0.3729; *p* = 0.0043). A very high positive correlation was also found between the concentration of MMP-8, and MMP-9 assessed in the peripheral blood serum (rho = 0.8387; *p* < 0.0001). A very high positive correlation was also observed in the case of MMP-8 and MMP-9 concentrations assessed in samples of cervical secretion from the posterior vaginal fornix (rho = 0.7798; *p* < 0.001). In the case of the remaining possible combinations of MMP-8 MMP-9 concentrations in peripheral blood serum and samples taken from the cervix, no statistically significant correlations were found. On the other hand, there were several correlations between demographic and clinical variables and the concentrations of MMP-8 and MMP-9 assessed in various study materials and an average negative correlation between MMP-8 values assessed in the cervical material and the age of the examined patients (rho = −0.4000; *p* = 0.0020). The age of the respondents also negatively correlated with the values of MMP-9 concentrations assessed in the material taken from the cervix (however, it was a weak correlation) (rho = −0.2986; *p* = 0.02). Similarly, a weak negative correlation was observed between the values of the parameters of the duration of pregnancy and MMP-9 (assessed in the material taken from the posterior vaginal vault) (rho = −0.3434; *p* = 0.0089).

Based on the data analysis, several correlations were observed between the selected demographic and clinical parameters in the control group. There was a statistically significant negative correlation between the values of SR parameters, comparative measurement of tissues, and cervical length (rho = −0.3743; *p* = 0.0416).

As in the study group, a high positive correlation was found between the concentration of MMP-8 and MMP-9 assessed in the peripheral blood serum (rho = 0.7511; *p* < 0.0001). A high positive correlation was also observed in the case of MMP-8 and MMP-9 concentrations assessed in samples of cervical secretion from the posterior vaginal fornix (rho = 0.5189; *p* = 0.0033). No statistically significant correlations were found for the remaining possible combinations of MMP-8 and MMP-9 concentrations in peripheral blood serum and samples taken from the posterior vaginal fornix.

There were, however, two correlations between clinical variables and the values of MMP-8 concentrations assessed in samples taken from the cervix.

Among other things, there was an average negative correlation between the MMP-8 values assessed in the cervical material and the SR parameter—comparative tissue measurement in the control group (rho = −0.3737; *p* = 0.0419). There was also an average negative correlation between MMP-8 values assessed in the material collected from the posterior vaginal fornix and the parameter—duration of pregnancy in weeks (rho = 0.3623; *p* = 0.0491). Based on the analysis carried out with the use of ROC curves, it was found that among the studied demographic and clinical variables, only the length of the cervix (in mm) and the concentration of MMP-8 (in ng/mL), assessed in the cervical secretion obtained from the posterior vaginal fornix, were significantly high in the area under the curve (AUC) value in the early detection of the possibility of preterm labor.

**Figure 6:**
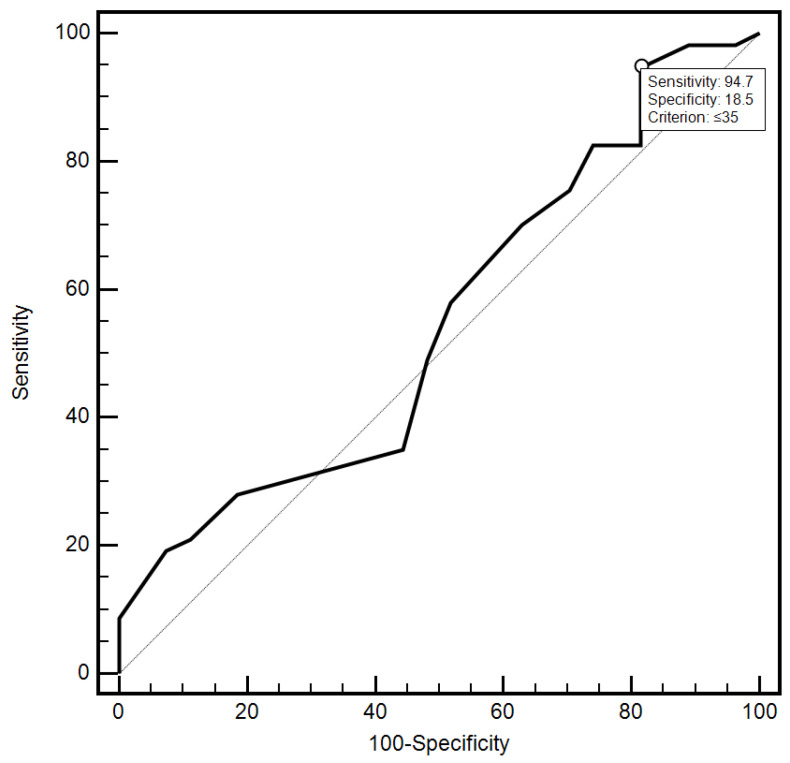
Diagnostic usefulness of the age parameter in the early detection of preterm labor using the ROC curve.

**Figure 7:**
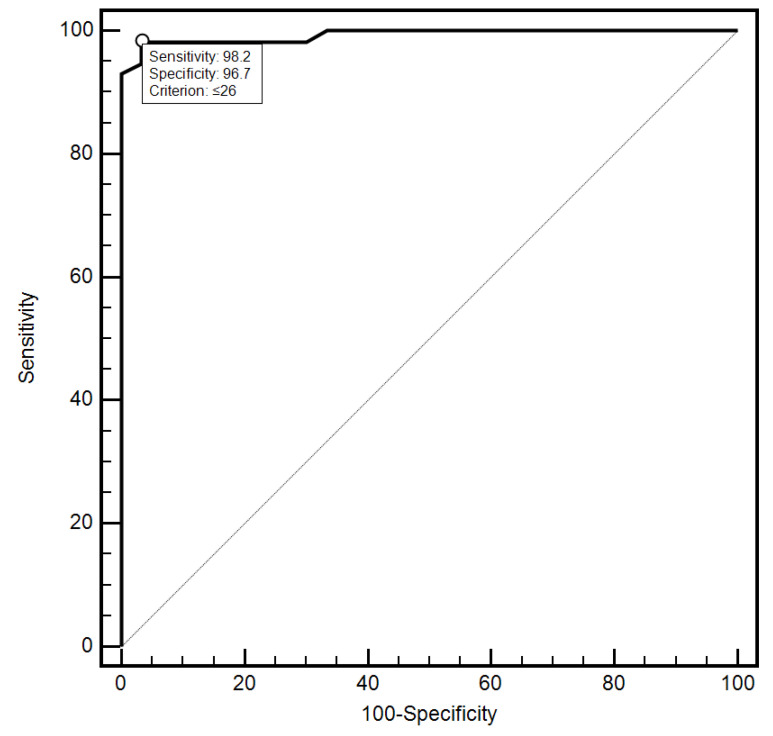
Assessment of the diagnostic usefulness of the cervical length parameter in early detection of preterm labor using the ROC curve.

**Figure 8:**
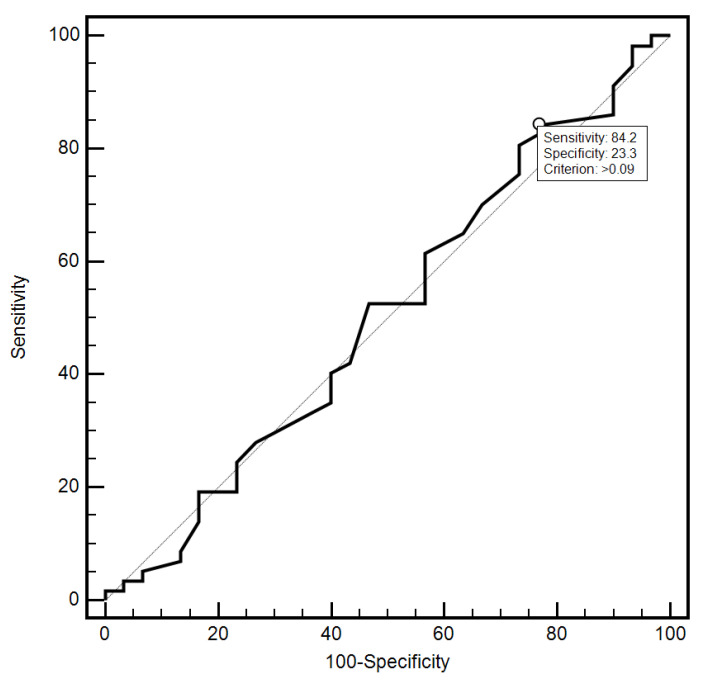
Evaluation of the diagnostic usefulness of the deformity parameter in the early detection of preterm labor using the ROC curve.

**Figure 9:**
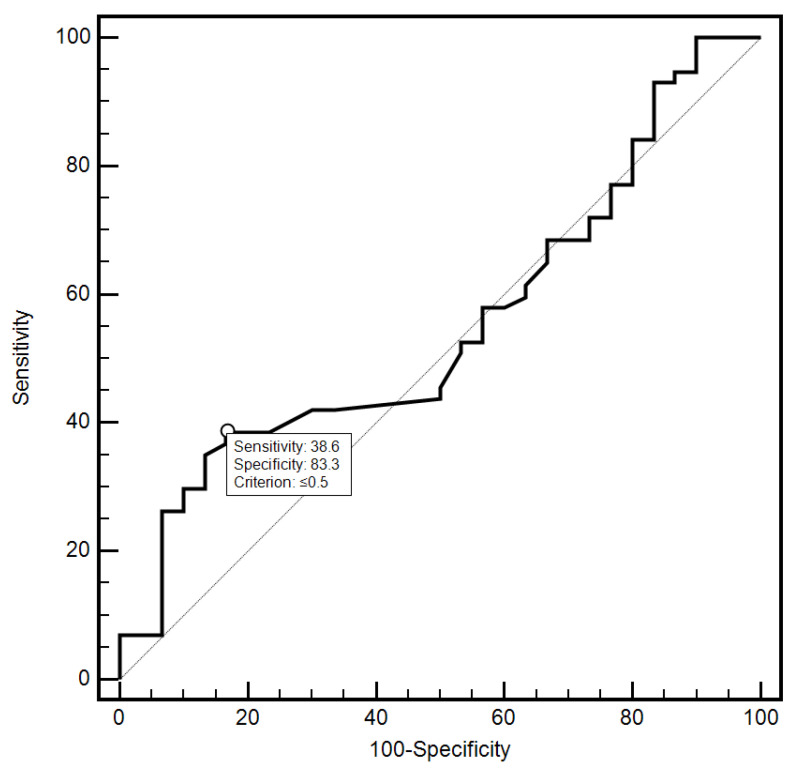
Diagnostic usefulness of the SR parameter—comparative tissue measurement in the early detection of preterm labor using the ROC curve.

**Figure 10:**
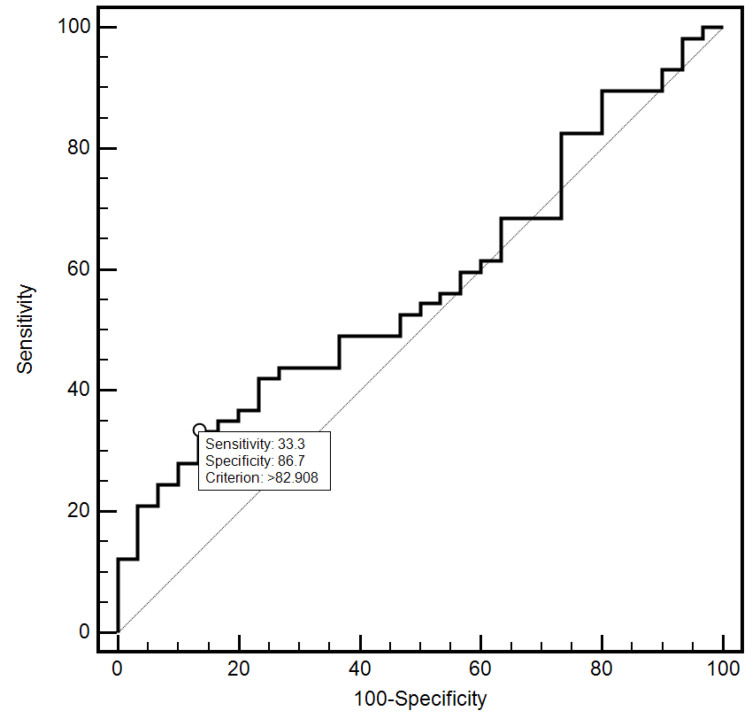
Assessment of the diagnostic usefulness of the MMP-8 (ng/mL) concentration determination in the peripheral blood serum in the early detection of preterm labor using the ROC curve.

**Figure 11:**
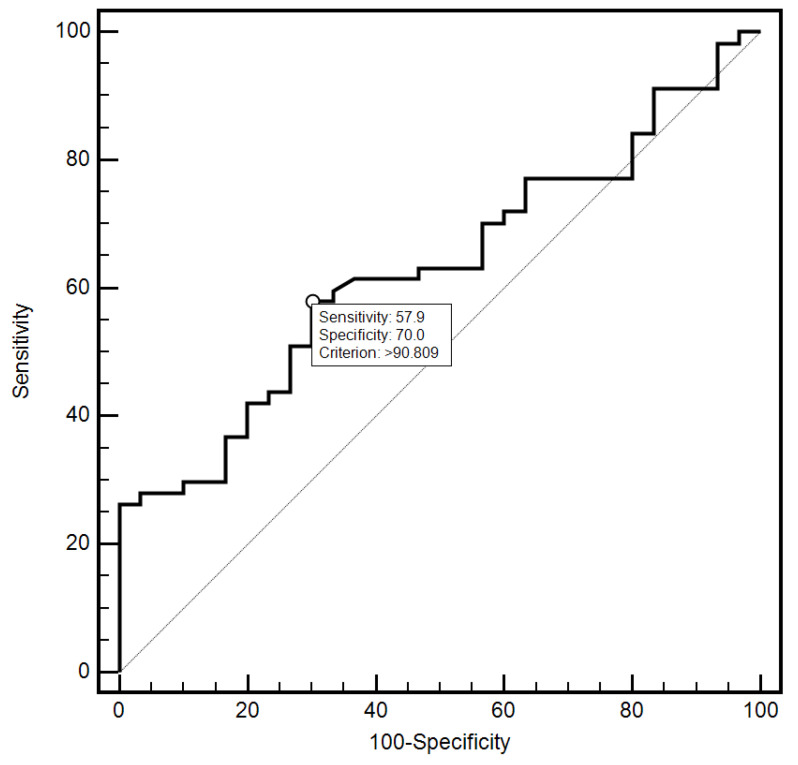
Evaluation of the diagnostic usefulness of MMP-8 (ng/mL) concentration determination in samples taken from the cervix in early detection of preterm labor using the ROC curve.

**Figure 12:**
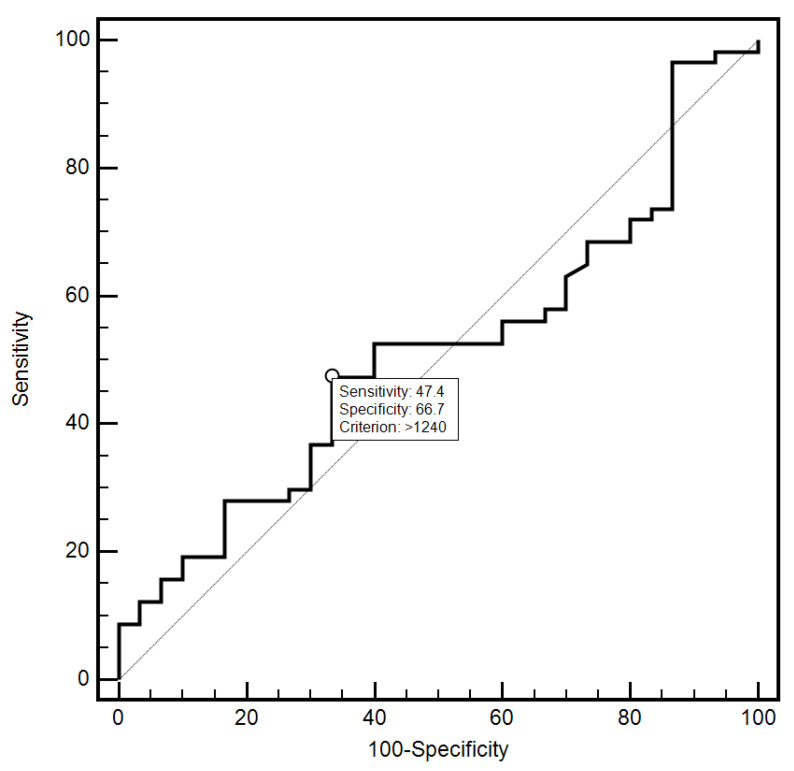
Assessment of the diagnostic usefulness of the MMP-9 (ng/mL) concentration determination in the peripheral blood serum in the early detection of preterm labor using the ROC curve.

**Figure 13:**
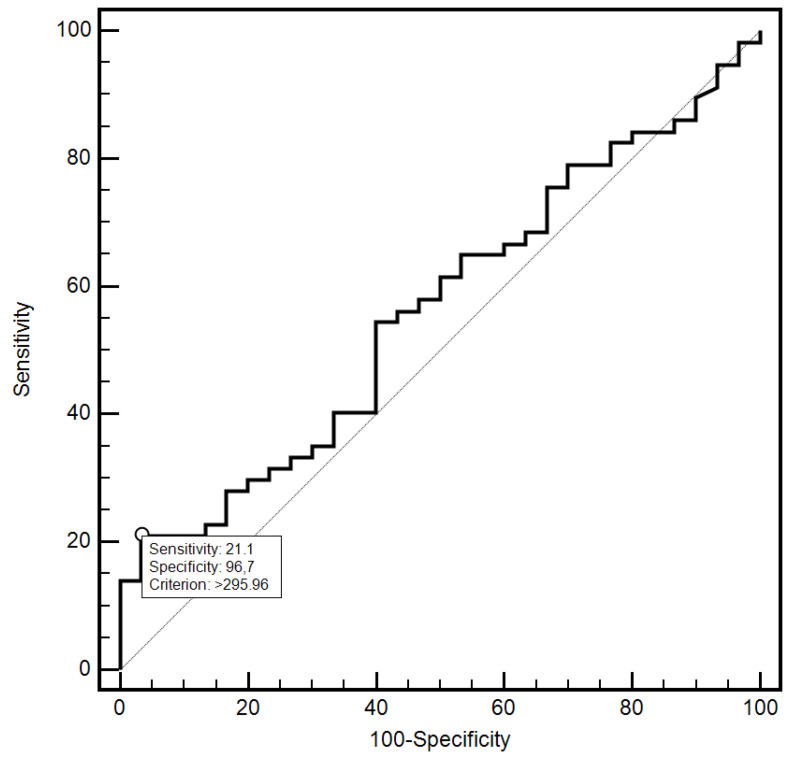
Evaluation of the diagnostic usefulness of MMP-9 (ng/mL) concentration determination in samples taken from the cervix in the early detection of preterm labor using the ROC curve.

For the cut-off value of the cervical parameter length ≤ 26 mm determined on the basis of the ROC curve, the sensitivity and specificity of the detection of preterm labor were 98.2% and 96.7%, respectively (AUC = 0.993, 95% CI: 0.437–0.659; *p* < 0.0001; Figure 6). On the other hand, in the case of the cut-off value for MMP-8 concentration assessed in peripheral blood serum > 90.8 ng/mL, the sensitivity and specificity of preterm labor detection were 57.9% and 70%, respectively (AUC = 0.631, 95% CI: 0.520–0.732; *p* < 0.0291; Figure 7). On the other hand, the other examined variables (sensitivity and specificity, respectively): age (94.7% and 18.5%), strain (84.2% and 23.3%), SR—comparative tissue measurement (38.6% and 63, 3%), concentrations of MMP-8 (33.3% and 86.7%) and MMP-9 (47.4% and 66.7%) assessed in peripheral blood serum, and the concentration of MMP-9 (21.1% and 96.7%), tested in samples from the cervix, proved to be insignificantly diagnostic in detecting preterm labor (Figure 6, Figure 7, Figure 8, Figure 9, Figure 10, Figure 11, Figure 12 and Figure 13). Detailed data on the assessment of the diagnostic usefulness of selected demographic and clinical factors using ROC curves are presented in Table 4.

## 5. Discussion

Despite a significant development that has taken place in recent years in medicine, preterm labor still remains one of the major problems of modern obstetrics and therefore is one of the most serious challenges of modern perinatology (1). The aim of this study was to assess the effects of biochemical markers and ultrasound measurement on predicting preterm labor. The physical markers included the assessment of cervical length and its elastographic assessment, while the biochemical factors included the assessment of the concentration of metalloproteinases in the vaginal-cervical secretion and in the maternal blood serum. Statistical analysis of the age of the patients did not show statistically significant differences. The test and control groups were homogeneous in terms of age. In the study group, the mean age was 29.7 years. However, in the control group, the mean age was slightly higher, amounting to 30.7 years). The age of the patients had no significant effect on the increased risk of preterm labor. Most of the scientific reports, analyzing the data on patients with extreme age, confirm the fact that the age of the patients influences the risk of preterm labor [26,27]. In a study by Cooper et al., obstetric results were assessed in very young 15-year-old married women with secondary education, white race, under medical care [26]. Similarly, Astolfi et al. Comparing the age of parents at 20–29 years with the age of the mother over 30 and the father over 40 showed that the influence of paternal age on the risk of preterm labor is smaller but significant if he is over 40 [28]. It is noteworthy that the deformation parameter (cervical) was slightly higher in older patients, but this relationship did not become statistically significant.

In this research, the variable “duration of pregnancy” in the study and control group was compared with the variable “cervical length” assessed in the ultrasound examination. It was confirmed that the parameter “duration of pregnancy in weeks” had significantly higher values in the control group compared to the study group. It was shown that in patients whose variable “cervical length” was less than or equal to 25 mm, the parameter “duration of pregnancy” in weeks had statistically significantly lower values. The obtained results lead to the conclusion that the CL expressed in millimeters is inversely proportional to the risk of preterm labor. This is confirmed by the Berghella study, according to which screening the length of the cervix with transvaginal ultrasound is a good prognostic test for the prediction of spontaneous preterm labor in single pregnancies with symptoms of preterm labor [29]. It has been shown that in patients at risk of preterm labor, for whom the cervical length measurement is known, the incidence of spontaneous preterm labor is lower, and the gestational age at delivery is higher. This is due to the possibility of using appropriate clinical management. Asymptomatic women at risk should be screened at a 2-week interval starting from 16 to 18 weeks, up to 24 weeks. CLs < 10th centile are at risk of PTB, especially with a decrease in CL after 16 weeks [30]

In our study, the results of the ultrasound assessment of the CL and an elastographic examination were analyzed. The evaluation of the cervical length measurement in relation to other parameters is the subject of many studies comparing the palpation with ultrasound examination of the cervix, especially in patients with premature contractions of the uterine muscle and preserved amniotic fluid [31,32,33,34]. Women between 24 and 34 weeks of pregnancy were assessed. On admission to the hospital, the cervix was assessed in relation to the Bishop scale and the length of the cervix measured by ultrasound in all patients. Statistical analysis showed a difference based on cervical evaluation methods. More medical information was provided by the results of ultrasound examinations. However, both methods had a similar predictive value [32]. In our study, the cervix was not assessed according to the Bishop score due to the subjectivity of this method, emphasized by other authors [33,34]. Similar results were obtained in the Pedretti screening [35]. It assessed the length of the cervix in ultrasound in asymptomatic patients. It has been confirmed that this form of examination can be successfully used to identify asymptomatic patients with single pregnancies at risk of preterm labor.

The aim of this study was to confirm the hypothesis of the existence of a correlation between the so-called “Soft cervix” in the elastographic evaluation and its reduced length, and therefore the use of elastography as a marker of premature labor. One of the inspirations for this assumption was a study conducted by Thomas et al. in 2007 in non-pregnant women, who assessed the elasticity of neoplastic changes in the cervix and, for the first time, used the elastographic technique to assess the cervix [36]. Thomas et al. created a cervical pattern. Measurements were made with a transvaginal probe with slight compression of the cervix. The elastographic images were analyzed using a program that allows semi-quantitative tissue stiffness analysis. Tissue elasticity distribution calculations were performed in real-time and presented using a color scale—red (soft), blue (hard), and green (medium-hard). Based on this study, it was determined that cervical neoplastic changes can be identified as less flexible structures within the cervix. The hypothesis of our research was based on the assumption that there is a dependence of cervical elasticity on the extent of pregnancy. Unfortunately, the evaluation of the correlation of parameters such as strain and SR, i.e., a comparative measurement of the tissues determining the consistency of the cervix with the “duration of pregnancy”, proved to be statistically insignificant. Therefore, the above-mentioned parameters are not good markers of preterm labor. The results on the basis of which this conclusion was drawn were calculated based on the analysis performed with the use of ROC curves. Similar research results were obtained by Maurer et al., who showed a very weak correlation between elastography and clinical features of the population, such as a history of preterm labor and cervical length less than 30 mm [37]. Due to the non-standardized values of the force exerted by the probe on the cervix, only an assessment of the relative formability within each organ can be obtained. Therefore, this study does not provide information about the evolution of changes in the elasticity of the entire organ because it does not lead to the normalization of the strength as the color scale signal depends on the imaged deformation, i.e., the susceptibility of the examined tissues. Based on the tests performed with the Voluson E8 apparatus, using the RIC5-9-D endovaginal probe with static elastography software, it was impossible to obtain the same tissue deformation forces during one ultrasound examination with the use of elastography, which conditioned the obtaining of comparable results, even when examined by the same person. Therefore, in the case of the calculated coefficients, mean values were taken into account, which were included in the statistical calculations. Fruscalzo et al. proposed standardization of the ROI area and developed guidelines for controlling the effect of a force applied with a transvaginal probe [38,39]. The ROI was placed across the entire thickness of the cervical anterior lip to reduce the variation due to tissue heterogeneity and force dispersion due to the distance from the transvaginal probe. Thus, it was possible to quantify cervical deformity on a continuous scale of values. In turn, Parra-Saavedra et al. calculated the cervical consistency index (CCI). In his study, he used the anteroposterior thickness of the cervix (AP), measured before and after applying pressure to the cervix, respectively [40]. A significant relationship between CCI and the elastographic assessment of the cervix was also confirmed in the study by Mazza et al. [34]. The above-mentioned authors emphasize that the deformation coefficient depends on the distance between the ROI frame and the transvaginal probe, i.e., the greater the elasticity of the test center, the less repeatable the test is. Therefore, in our study, the reference point of reference was the hard parts of the fetus (skull bones), considering them as reference areas in relation to the cervix due to similar bone mineralization in a given week of pregnancy. The results of our own research showed an identical deformation coefficient for both groups. On the other hand, SR, i.e., the comparative tissue measurement, differed slightly between the studied groups but did not reach the level of statistical significance. The obtained results did not show a relationship between tissue elasticity and the duration of pregnancy and did not confirm the hypothesis that the less elastic the cervical tissue, the lower the risk of preterm labor.

Promising research, opposed to our own research, was Nicolaides’s group’s 2012 analysis. Its purpose was to confirm the objectivity of the elastographic examination. The repeatability of elastographic measurements was then assessed. Two investigators assessed the cervixes of the same pregnant women. No statistically significant differences were found in the elastographic measurements by both researchers, except for the area that directly perceived the power of the transducer. On the basis of the conducted analysis, it was concluded that an objective assessment of the cervical elastogram is possible [33]. It seems, however, that the elastographic evaluation of the neck has some limitations, as the studies conducted by the author did not confirm the conclusions resulting from the above-mentioned analyzes. No statistically significant correlation was found between the value of the parameters assessed and the length of the cervix and the duration of pregnancy; therefore, the usefulness of elastography in a clinical study in predicting preterm labor was not demonstrated. Moreover, in the study group, a negative correlation between the values of deformation and the SR measurement of tissues was noted, and in the control group, a negative correlation between the values of SR parameters—comparative measurement of tissues and the length of the cervix. This did not allow for a clear comparison of the two groups in terms of the above-mentioned correlations and thus to draw conclusions about the usefulness of this study in predicting preterm labor.

Further studies are needed to evaluate the suitability of this technique for clinical application, such as predicting preterm labor or successful induction of labor. In our own study, the elastographic evaluation used a numerical scale obtained in the Elastography Analysis program. However, elastographic maps in terms of color were not assessed, as was done, for instance by Woźniak et al. [41]. The above-mentioned researchers found that elastographic evaluation of the internal cervix in 18–22 weeks of pregnancy in patients with shortened cervix may be useful in predicting premature labor. The hardness of the inner orifice of the cervix was assessed elastographically using a color scale: red (soft), yellow (medium-soft), blue (medium-hard), and purple (hard). In the case of visualization of two colors around the inner mouth, the softer option was chosen. The following variables were analyzed: the percentage of premature births in the different categories of internal orifice hardness and the sensitivity, specificity, negative and positive predictive value of elastography in predicting preterm labor. The number of premature deliveries was significantly higher in the red group than in the blue and purple groups. The cut-off point for elastography suggests adopting both red and yellow colors as factors predisposing to preterm labor. The comparison of both methods (elastographic map and numerical scale) is not really possible. The parameters assessed in the own study (deformation and SR—comparative tissue measurement) in the study group showed a statistically significant average negative correlation with each other, which does not allow for drawing unequivocal conclusions. No results were obtained that would confirm the conclusions formulated by the authors. However, it should be taken into account that the methods used by the author differed radically from the methods used by Woźniak et al., as can be seen, elastography seems to be a promising diagnostic method.

Unfortunately, there is still no consensus on the optimal method for assessing the cervix. Virtually most of the previous studies, evaluating the use of various elastography methods, gave many satisfactory results [42,43,44].

In this study, the relationship between the concentration of metalloproteinases in blood serum and vaginal-cervical secretions in pregnant women was analyzed. Most studies analyze the concentration of MMP-8 and MMP-9 in the amniotic fluid in association with other fluid components and their correlation with preterm labor. The latest one aimed at investigating the association of MMP-1, MMP-8 and MMP-9 polymorphisms, and levels of MMP-9 in preterm birth with positive results [45]. A study by Lee et al. found that a model combining the concentration of various amniotic fluid proteins, including MMP-8 and MMP-9, with clinical factors can improve the accuracy of preterm labor prediction [46]. Moreover, the assessment of these correlations is more accurate than the assessment of single biomarkers in women with cervical insufficiency. The concentration of MMP-8 in the cervical secretion was significantly higher in the study group as compared to the control group. Therefore, it can be concluded that the measurement of MMP-8 concentration can be one of the methods of predicting preterm labor, as was stated in research by Lee and Park [47]. Yoo et al. also assessed the concentration of metalloproteinases in the cervicovaginal secretion. He showed that proteins involved in immune regulation, including MMP-8 and MMP-9, alone or in combination with clinical risk factors, may be useful as predictors of spontaneous preterm labor in women with cervical insufficiency or with an ultrasound short cervix (≤25 mm) [48]. The combination of these markers and clinical factors significantly improves the predictability of preterm labor compared to the markers alone. The results obtained in our own study confirm the above-mentioned concept. A statistically significant positive correlation was found between the cervical length values and the duration of pregnancy, and it was also confirmed that the concentration of MMP-8 in the uterine cervical discharge is significantly higher in the study group compared to the control group. Therefore, it can be concluded that measuring the concentration of MMP-8 together with the evaluation of the cervix may be useful markers of preterm labor.

Scientific studies have shown that in pregnancy, the concentration of matrix metalloproteinases in the cervical mucus is statistically higher [49]. However, their physiological and pathophysiological significance is not fully understood and elucidated, although it has been proven that the concentrations of MMP-8 and MMP-9 increase mainly in the distal part of the mucous plug depending on the stages of pregnancy. It seems that it is related to the defense against infectious agents acting mainly in this area of the cervix. In patients delivering prematurely, the concentrations of MMP-8, MMP-9, and IL-8 in the cervical secretion are several times higher compared to the concentrations of these substances in the mucous plugs of patients delivering at term. As there are different molecular mechanisms underlying preterm labor without and from damage to the membranes, the concentration of MMP-8 in the cervical secretion may thus reflect the different functions of this protease. Therefore, the use of MMP-8 to differentiate the causes of preterm labor is not recommended [50]. However, it does not change the fact that MMP-8 can be effectively used as a marker of premature labor.

In our study, the group of patients was quite heterogeneous in terms of the cause of preterm labor, and yet in all patients, the concentration of this metalloproteinase was significantly increased in the cervical secretion. On the other hand, the concentration of metalloproteinases in the peripheral blood serum of this dissertation is consistent with the results obtained by other researchers and does not show significant differences between patients with a physiological pregnancy and patients with a risk of premature delivery [51]. In this study, the concentrations of MMP-9 in the blood serum and in the cervical secretion were assessed. It would seem that the concentrations of MMP-9 should statistically significantly differ between the study group and the control group. Unfortunately, the tested material did not provide any results confirming this thesis. The concentration of MMP-9 in the blood serum in patients in the study group did not differ significantly from the concentration of MMP-9 in the control group, although the difference between the concentration in the cervical secretion collected from the posterior vaginal fornix in the study group was slightly higher, but not statistically significant. Athayde et al. conducted a study to determine whether the increased bioavailability of MMP-9 was associated with preterm labor [52]. The results were as follows: spontaneous delivery at term was associated with a statistically significant increase, and the concentration of MMP-9 in the amniotic fluid was statistically higher in the group of women with preterm labor compared to the group of women with the risk of preterm delivery, who gave birth at the expected date of delivery. Moreover, MMP-9 concentrations did not change with the progressive gestational age. In conclusion, a significant increase in MMP-9 concentration is characteristic of PPROM (preterm premature rupture of membranes); therefore, MMP-9 cannot be considered a marker of preterm labor. This concept is also confirmed by the results of our study. The study conducted by the authors showed differences in the concentrations of both metalloproteinases in the study group as compared to the control group; however, both groups were not analyzed in terms of the etiopathogenetic factor of preterm labor, i.e., infection. The concentrations of MMP-8 and MMP-9 in the cervical secretion were higher in patients in the study group; the concentrations of MMP-8 were significantly higher while the concentrations of MMP-9 differed slightly. Similar results were obtained by Myntti et al., but in his study, the cause of preterm labor was important [53]. This study assessed the proteolytic biomarkers of the amniotic fluid that form the inflammatory cascade in response to microbial invasion of the amniotic cavity and intrauterine infection in preterm labor with intact membranes. The research results obtained by the author indicate that the concentrations of MMP-8, MMP-9 (and others) were higher in the case of suspected infection compared to the control group and also in the case of intrauterine infection. The tested biomarkers, including MMP-8 and MMP-9, had a sensitivity of 100% with thresholds based on the ROC curve. The analysis of the study group indicates that in some patients, the cause of preterm labor could be an intrauterine infection.

Metalloproteinases are involved in the pathogenesis of preterm labor but are not only related to intrauterine birth. Therefore, an increase in their concentrations may be an important predictive factor, especially in the case of MMP-8 not related to the infectious nature of preterm labor. The results of the authors’ research indicate that the monitoring of the length of the cervix and the non-invasive assessment of biochemical markers of preterm labor, including metalloproteinases, may allow for the personalization of pregnancies at risk of preterm labor and, in the long term, also enable effective prevention of a number of serious perinatal complications. Delivery and proper postnatal care in properly equipped perinatal centers, as well as timely implemented therapeutic interventions, can significantly reduce the incidence of preterm labor and the effects of prematurity.

## 6. Conclusions

Assessment of the cervical length plays a key role in the ultrasound assessment of cervical length and is one of the most important markers of preterm labor. The use of static elastography does not meet the criterion of a good marker of premature labor in high-risk patients. However, in the same group of patients, the MMP-8 concentration may be one of the methods of predicting preterm labor.

## Figures and Tables

**Table 1:** Demographic, socio-clinical characteristics of the study and control group.

Parameter		Study No (*n*)	Control No (*n*)	Study Percentage (%)	Control Percentage (%)
Age	20–30 yo	38	19	63..33	66.67
	31–40 yo	19	11	36.67	33.33
Education	Secondary	10	1	3.33	17.54
	Higher	47	6	20	82.46
Place of living	Rural	18	23	76.67	31.58
	Urban < 100,000 people	14	11	36.67	24.56
	Urban > 100,000 people	25	6	20	43.86
Employment	Employed	28	13	43.33	49.12
	Unemployed	29	16	53.33	5.88
Number of pregnancies	1	37	14	46.67	64.91
	2	14	16	53.33	24.56
	3	4	9	30	7.02
	4	2	3	10	3.51
Number of deliveries	1	41	2	6.67	71.93
	2	15	17	56.67	26.32
	3	1	11	36.67	1.75
High-risk factors	N/A	29	2	6.67	50.88
	PTB in family	7	18	60	12.28
	Infertility	6	1	3.33	10.53
	Miscarriage	6	3	10	10.53
	PTB	3	1	3.33	5.26
	Cervical incompetence	3	2	6.67	5.26
	Urinary and vaginal tract infection	3	2	6.67	5.26
	FGR		1		3.33
	Hypothyroidism		1		3.33
	Uterine myomas		1		3.33

Abbreviations: yo—year old, N/A—not applicable, PTB—preterm birth, FGR—Fetal Growth Restriction.

**Table 2:** Detailed characteristics of the study group in terms of selected demographic and clinical parameters.

Parameter	N	Average	Median	Min	Max	SD
Age (years) *	57	29.70	30	22	39	3.93
Duration of gestation (wks)	57	36.79	38	25	41	4.10
Length of cervix (mm)	57	17.49	19	2	30	6.34
Deformation	57	0.20	0.17	0.01	0.94	0.14
SR-comparative tissue measurement	57	0.83	0.59	0.03	3.86	0.77
MMP-8 serum (ng/mL)	57	74.17	61.63	16.59	287.77	51.50
MMP-8 cervix (ng/mL)	57	155.46	158.34	2.07	379.58	123.14
MMP-9 serum (ng/mL)	57	1308.98	1181.90	418.94	2569.52	591.88
MMP-9 cervix (ng/mL)	57	202.43	103.04	1.72	1258.57	281.85

*—data with a normal distribution (assessed with the Shapiro–Wilk test). N—number of cases, SD—standard deviation

**Table 3:** Detailed characteristics of the control group in terms of selected demographic and clinical parameters.

Parameter	No	Average	Median	Min	Max	SD
Age (years) *	27	30.74	30	25	39	4.07
Duration of gestation (wks)	30	39.10	40	28	41	2.26
Length of cervix * (mm)	30	34.73	34	25	46	6.37
Deformation	30	0.20	0.16	0.009	0.61	0.13
SR—comparative tissue measurement	30	1.23	0.57	0.17	6.56	1.65
MMP-8 serum (ng/mL) *	30	58.44	57.47	14.99	123.73	26.22
MMP-8 cervix (ng/mL)	30	94.19	60.95	1.14	259.02	91.08
MMP-9 serum (ng/mL) *	30	1261.56	1134.42	447.30	2258.83	505.83
MMP-9 cervix(ng/mL)	30	103.59	59.70	2.24	554.40	122.21

*—data with a normal distribution (assessed with the Shapiro–Wilk test). WKS—weeks gestation

**Table 4:** Characteristics of the parameters of ROC curves for the concentration values of MMP-8 cervical (ng/mL).

Parameter	AUC	SE	95%CI	Level (AUC = 0.5)
Age (years)	0.550	0.068	0.437–0.659	0.4674
Cervical length (mm)	0.993	0.006	0.945–1.000	<0.0001
Deformation	0.506	0.067	0.397–0.615	0.9240
SR-comparative tissue measurement	0.553	0.064	0.443–0.660	0.4067
MMP-8 cervix (ng/mL)	0.631	0.059	0.520–0.732	0.0291
MMP-8 serum (ng/mL)	0.575	0.062	0.465–0.681	0.2275
MMP-9 cervix (ng/mL)	0.562	0.063	0.452–0.668	0.3266
MMP-9 serum (ng/mL)	0.514	0.064	0.405–0.623	0.8236

AUC—Area Under the Curve, SE—standard error, CI—confidence interval.
